# Update on the Pathophysiological Activities of the Cardiac Molecule Cardiotrophin-1 in Obesity

**DOI:** 10.1155/2013/370715

**Published:** 2013-04-10

**Authors:** Mohamed Asrih, François Mach, Alessandra Quercioli, Franco Dallegri, Fabrizio Montecucco

**Affiliations:** ^1^Division of Cardiology, Faculty of Medicine, University of Geneva and Geneva University Hospital, Foundation for Medical Researches, Avenue de la Roseraie 64, 1211 Geneva 4, Switzerland; ^2^Department of Internal Medicine, First Clinic of Internal Medicine, University of Genoa, 6 Viale Benedetto XV, 16143 Genoa, Italy

## Abstract

Cardiotrophin-1 (CT-1) is a heart-targeting cytokine that has been reported to exert a variety of activities also in other organs such as the liver, adipose tissue, and atherosclerotic arteries. CT-1 has been shown to induce these effects via binding to a transmembrane receptor, comprising the leukaemia inhibitory factor receptor (LIFR**β**) subunit and the glycoprotein 130 (gp130, a common signal transducer). Both local and systemic concentrations of CT-1 have been shown to potentially play a critical role in obesity. For instance, CT-1 plasma concentrations have been shown to be increased in metabolic syndrome (a cluster disease including obesity) probably due to adipose tissue overexpression. Interestingly, treatment with exogenous CT-1 has been shown to improve lipid and glucose metabolism in animal models of obesity. These benefits might suggest a potential therapeutic role for CT-1. However, beyond its beneficial properties, CT-1 has been also shown to induce some adverse effects, such as cardiac hypertrophy and adipose tissue inflammation. Although scientific evidence is still needed, CT-1 might be considered as a potential example of damage/danger-associated molecular pattern (DAMP) in obesity-related cardiovascular diseases. In this narrative review, we aimed at discussing and updating evidence from basic research on the pathophysiological and potential therapeutic roles of CT-1 in obesity.

## 1. Introduction 

Since its recent recognition as a disease [1], the scientific community started to consider obesity as a disorder reaching epidemic proportions in developed countries and bearing an increased cardiovascular risk [2, 3]. Intensive investigations on the pathogenesis of obesity have been performed to better understand the molecular mechanisms underlying the disease development and its association with atherogenesis. Therefore, novel molecules have been identified as potential common mediators influencing obesity, atherosclerosis as well as postinfarction tissue injury. In particular, recent evidence suggests that endogenous molecules (also named damage/danger-associated molecular patterns (DAMPs)) might interfere with both innate and adaptive immunity as well as atherosclerotic inflammation in several phases of the disease [4]. For instance, some adipocytokines (such as adiponectin, leptin, or resistin), which have been shown to be dysregulated in obese subjects [5], have been suggested as critical cardiovascular risk biomarkers in atherosclerotic diseases [6, 7]. On the other hand, considering that gp130 ligands have been shown to modulate the energy balance in obesity [8], these molecules have been investigated as a potential therapeutic targets against insulin resistance [8, 9]. Cardiotrophin-1 (CT-1) is a gp130 ligand and a member of the interleukin- (IL-) 6 family, originally described as an active inducer of cardiac hypertrophy, atherosclerosis and, thus, a potential appropriate example of DAMP [10]. *In vivo *studies indicated that CT-1 induced hypertrophic properties within the myocardium [11]. In fact, chronic systemic administration of CT-1 (up to 2 microg twice a day for 14 days) dose dependently induced cardiac hypertrophy in mice (assessed as an increase in the ventricular weight without an increased number of cardiomyocytes) [12]. In addition to hypertrophy, increased circulating plasma levels of CT-1 have been associated with the development of the metabolic syndrome [13]. Consistently, chronic exposure of adipocytes to CT-1 resulted in decreased insulin responsiveness [14]. Confirming previous studies that identified CT-1 as an highly protective molecule in many tissues (such as the kidney and the liver) [15–17], a recent study showed that acute and chronic treatments with recombinant CT-1 were able to correct insulin resistance in animal models of genetic and acquired obesity [18]. In this narrative review, we aimed at discussing the activities of CT-1 and its potential implications as a therapeutic molecule in obesity. In addition, the specific role of CT-1 as a DAMP directly triggering atherosclerotic and adipose tissue inflammation will be updated.

## 2. Potential Sources of Cardiotrophin-1 

The expression of CT-1 has been originally identified on neonatal cardiomyocytes [10]. In addition, CT-1 transcripts have also been detected in many other tissues [19, 20]. Interestingly, a recent study revealed that during the first period of the embryonic development (day 8.5), CT-1 is confined in the primitive heart tube in mice [20]. During the heart development, CT-1 is expressed within the atriums and ventricles, but not in the endocardium. At a later developmental stage, CT-1 is expressed at relatively high level within the global heart, whereas most of the other organs (such as brain, liver, kidney, and lung) have been shown to express relatively low protein levels of CT-1 [20]. These studies indicate that CT-1 might play a fundamental role in the cardiac development. Indeed, this is supported by the study of Yoshida and coworkers showing that gp130 (a subunit of the CT-1 receptor) knockout mice develop myocardial ventricular hypoplasia, resulting in death in utero [21]. In adult animals, gp130 is ubiquitously expressed suggesting a physiological role of this receptor not only during embryogenesis but also during adult life. Consistently, the postnatal inactivation of gp130 leads to severe defects in both the heart and other systems in mice [22]. Mice lacking specifically cardiac functional gp130 during both embryonic and adult periods have been shown to develop normal cardiac structure and function [23]. However, in response to an aortic pressure overload, these conditional knockout mice have been observed to be rapidly affected by a dilated cardiomyopathy with increased cardiomyocyte apoptosis when compared to the control mice [23]. Although it is important to note that CT-1 is mainly synthesized within the heart by both cardiomyocytes and noncardiac cells, the cardiac defects observed in gp130 knockout mice may be partially associated with disorders in the other animal organs with a final negative impact on the myocardium. 

Once produced by the heart, CT-1 is secreted through the coronary sinus into the peripheral systemic circulation [24]. Similar to the myocardium, CT-1 has been shown to be expressed by many other tissues such as adipose tissue, liver, lung, and skeletal muscles [12]. For instance, adipocytes have also been shown as an important cellular source of CT-1 in both physiological and pathological conditions [13]. On the other hand, increased levels of CT-1 have been detected in a variety of pathological states, where this mediator might influence the tissue function and injury [13, 25, 26]. For instance, CT-1 has been found to be upregulated in the hypertrophic ventricles of genetically hypertensive rats [27]. Similarly, enhanced CT-1 expression has also been reported in the rat ventricle after myocardial infarction [28]. 

CT-1 expression has been shown to be regulated by various transcription factors that are activated only under pathophysiological conditions, such as hypoxia [29, 30]. Both systemic and local levels of CT-1 might be increased as a result of not only an upregulated production, but also a potential reduction in its degradation. However, no evidence is currently available on the potential catabolic pathways of CT-1. One of the major proteolytic structures for cytosolic proteins is represented by proteasome [31]. Since it has been shown that nonobese diabetic mice might be characterized by a defective proteasome activity [32], modifications in CT-1 circulating and tissue levels could be explained by a reduction of the proteasome-mediated catabolism. However, this point remains highly speculative and requires further investigations. In addition, other traditional catabolic pathways for CT-1 elimination have been never explored. This approach might represent an interesting research field to better understand the dynamics and activities of CT-1 in cardiovascular diseases and obesity.

## 3. Regulation of CT-1 Expression

Since CT-1 was originally identified as a cytokine inducing cardiac hypertrophy, several basic research studies (targeting the regulation of the CT-1 gene expression *in vitro* in cardiomyocytes and rodent cardiovascular disease models) have been published. Similar to the human gene [19], mouse CT-1 was shown to contain several consensus sequences, such as SP-1, CRE, NF-IL6, AP-1, AP-2, and GATA [20]. Since CT-1 has been proposed to prevent the cardiac ischemic injury, a recent study showed that the hypoxia-inducible factor 1 (HIF-1) markedly enhanced the expression of CT-1, resulting in an improved cardiomyocyte survival in response to ischemia [29]. Others found that 60-minute hypoxia upregulated the mRNA expression of CT-1 in primary cultured cardiomyocytes [33]. Furthermore, hypoxia concomitantly upregulated both CT-1 and HIF-1 mRNA and protein expression in embryonic stem-cell-derived cardiac cells, confirming a protective role with improvement of both cell survival and proliferation [34]. In addition, CT-1 expression might be regulated by endocrine factors such as norepinephrine [33], aldosterone [35], fibroblast growth factor-2 (FGF-2) [36], and urocortin [37]. For instance, FGF-2 has been shown to increase CT-1 mRNA levels within the heart. In this work, Jiang and coworkers demonstrated that intracardiac administration of FGF-2 (2 microg/heart) reduced the infarct size, and induced postinfarction hypertrophy in a rat model of acute myocardial infarction and irreversible chronic ischemia [36]. These effects were associated with the cardiac upregulation of the CT-1, suggesting that FGF-2 might directly modulate CT-1 expression.

On the other hand, aldosterone, which is a recognized inducer of cardiac hypertrophy [38], has also been shown to increase CT-1 expression in cultured HL-1 cardiomyocytes [35]. In this study, the authors demonstrated that aldosterone-mediated activation of mineralocorticoid receptors was associated with the upregulation of CT-1 expression via the phosphorylation of the cytosolic p38 mitogen activated protein kinase (MAPK) [35]. Since p38 MAPK activation was shown to regulate the expression of IL-24 by stabilization of the 3′UTR of IL-24 mRNA [39], we can speculate that a similar mechanism might also influence p38 MAPK-induced expression of CT-1. 

High glucose and insulin levels have been demonstrated to promote cardiac hypertrophy [40, 41]. More recently, Liu and coworkers showed that the antidiabetic drug pioglitazone was able to reduce glucose and insulin levels in diabetes and concomitantly inhibit *in vitro* cardiomyocyte hypertrophy. Importantly, the authors showed that both glucose- and insulin-induced myocardial hypertrophy might be mediated by CT-1, suggesting that CT-1 expression could be directly increased by insulin and glucose stimulations [42]. These studies have not been confirmed by other research groups and require additional validations to support a crucial pathophysiological role for CT-1 in the cardiac microenvironment. The different sources of CT-1 as well as the molecular mechanisms influencing its production remain to be clarified.

## 4. CT-1-Triggered Signaling Pathways

**Figure 1:**
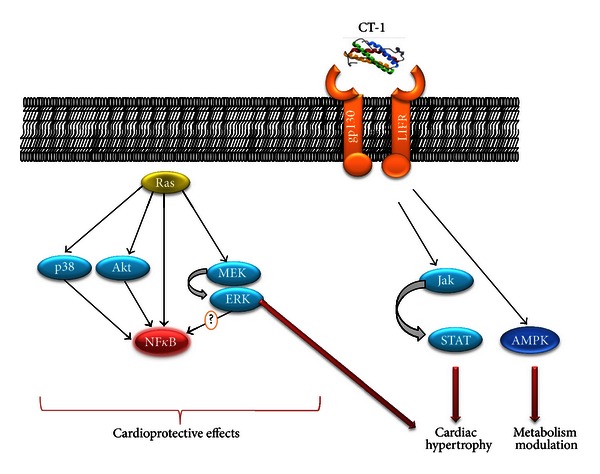
Activation of the Jak-Stat, MAP kinase signal transduction pathways, and AMPK, by CT-1 in cardiomyocytes. The mechanisms of Stat activation as well as a putative signaling cascade leading to NF*κ*B (nuclear factor-kappa B) activation are shown. Downstream target genes linking the Jak-Stat, MAP kinase signal transduction pathways, and AMPK to the induction of cardiomyocyte hypertrophy, antiapoptotic and metabolic effects of CT-1 remain to be elucidated.

CT-1-induced effects are mediated through the molecular binding to a transmembrane receptor gp130/leukaemia inhibitory factor receptor (LIFR) (Figure 1) [43]. This receptor is composed of two subunits (gp130 and LIFR) that are both necessary for an effective intracellular signal transduction [44]. Upon binding of CT-1 to its receptor, several signaling pathways have been shown to be activated. For instance, the antiapoptotic effects of CT-1 in cardiomyocytes are mediated by the activation of the p38 MAPK, protein kinase B (or Akt), and extracellular regulated kinases (ERKs) [45]. The downstream mechanisms involved in the cytoprotective role of these kinases remain controversial and are still under intensive investigation. Since several antiapoptotic signaling pathways mediate their effects through activation of the transcription factor NF*κ*B, CT-1 has been proposed to potentially activate NF*κ*B [45]. Confirming this hypothesis, CT-1-induced NF*κ*B activation and associated cardiomyocyte protection against hypoxic stress were abrogated using selective inhibitors of p38 MAPK, ERKs, or Akt in cultured adult rat cardiomyocytes [45]. This study indicates that intracellular kinase activation is required for CT-1-mediated benefits in cultured cardiomyocytes [45]. MEK5-ERK5 pathway has been also shown to be activated by CT-1 in cardiac hypertrophy [46]. In addition to these pathways, it has been demonstrated that CT-1 might modulate *in vitro* the activity and expression of the suppressor of cytokine signaling (SOCS3) as well as the peroxisome proliferator-activated receptor (PPAR) within cardiomyocytes [14, 18, 19].

Similar to other members of the IL-6 family, the binding of CT-1 to its receptor induces a plethora of intracellular signaling pathways that transduce its effects [11, 47]. Tian and coworkers have shown that the selective inhibition of signal transducer and activation of transcription- (STAT-) 3 strongly reduced CT-1-induced hypertrophy in cultured rat cardiomyocytes [48]. Importantly, ERK1/2 phosphorylation in this model was associated with the inhibition of STAT3 pathway, thus negatively regulating CT-1-mediated benefits [48]. The controversial results shown in the articles cited above mainly confirm that further investigations are needed to identify the different CT-1-triggered pathways.

## 5. Specific Role of CT-1 in Obesity 

Obesity is associated with a chronic low-grade inflammatory state, characterized by elevated circulating levels of cytokines, and the activation of proinflammatory signaling pathways [49, 50]. Wellen and Hotamisligil analyzed findings from several studies investigating metabolic and immunological disorders in obesity and related cardiovascular diseases [50]. The authors recommended considering systemic inflammation as a promoting factor for the metabolic and cardiovascular diseases [50]. Insulin resistance has been also described as strongly associated with abnormal accumulation of adipose tissue in obesity [51]. Therefore, adipocytes were investigated not only as an energy storing organ, but also as critical players in the regulation of glucose metabolism. More recently, it became evident that adipose tissue also represents a major source of hormones and cytokines (also called adipokines) [5]. In particular, Hotamisligil and coworkers confirmed that adipocytes display several immunomodulatory properties, including the secretion of inflammatory hormones and cytokines [52]. Importantly, these mediators have been shown as pivotal players in the modulation of inflammation, as well as glucose and lipid metabolism [50]. Among these molecules, the members of the interleukin-6 (IL-6) family (thus, potentially CT-1) have been directly correlated with development of insulin resistance in asymptomatic subjects [53] as well as in frankly diabetic patients [54]. In particular, CT-1 has been hypothesized to promote insulin resistance in cultured adipocytes [14]. The point that CT-1 might directly induce insulin resistance and it can be in turn upregulated by hyperglycemia or hyperinsulinemia represents a matter of debate. Chronic treatment with recombinant CT-1 has been shown to potentially downregulate food intake in mice [55]. In humans, intense physical exercise has been shown to be directly associated with plasma CT-1 levels [56]. This potential nutritional and stress-mediated rapid regulation of CT-1 levels is in partial contrast with the hypothesis of a causal activity of CT-1 in insulin resistance and potentially associated metabolic syndrome. Furthermore, it was found that the molecular pathways underlying such response involve an increased activation of STAT1, 3, 5A, and 5B, as well as the upregulated expression of SOCS3 mRNA in adipocytes [14]. Concomitantly, a transient decrease of the peroxisome proliferator-activated receptor *γ* (PPAR*γ*) mRNA was also observed in these cells [14]. The chronic stimulation with CT-1 on 3T3-L1 adipocytes also resulted in a decrease of both fatty acid synthase and insulin receptor substrate-1 protein expression [14]. Taken together, these results revealed that evidence for a direct role of CT-1 as potential promoter of insulin resistance in adipocytes remains to be confirmed [14]. However, this study did not reveal whether adipocytes are a major source of CT-1 and whether circulating CT-1 is enhanced in patients with an increased adipose tissue mass. Accordingly to the known association of centripetal obesity with other cardiovascular risk factors (i.e., in metabolic syndrome) [57], increased circulating levels of CT-1 have been also confirmed in patients with obesity and hyperglycemia as compared to healthy controls [13]. Indeed, Natal and coworkers showed that adipocytes under hyperglycemic conditions were an important source of CT-1, which might in turn favor insulin resistance [13]. Different from adults, obesity in adolescents has not been confirmed as a condition characterized by increased plasma levels of CT-1 [58]. In particular, also body mass index and waist circumference did not correlate with CT-1 plasma levels in adolescents [58]. Importantly, in contrast to adults, systolic blood pressure at rest correlates inversely with CT-1 [58], suggesting that CT-1 might not be a pathophysiological link between obesity, insulin resistance and metabolic syndrome in young human beings. Considering these controversial results in humans and mice, it could not be concluded that CT-1 might directly promote insulin resistance, particularly in hyperglycemic and obese patients [13]. Evidence from studies in animal models is in partial contrast with these preliminary results *in vitro* and in human beings. Surprisingly, CT-1 knockout mice were shown to develop insulin resistance that could be prevented by administration of exogenous CT-1 [18]. Indeed, the same CT-1 knockout mice also developed dyslipidemia, hypercholesterolemia, type 2 diabetes, and adult onset obesity, thus mimicking the human metabolic syndrome. However, in contrast to humans where metabolic syndrome is often associated with an increase in food intake, in these animals, metabolic defects result from reduced energy expenditure [18]. Exogenous CT-1 administration in wild-type mice increased energy expenditure, fatty acid oxidation, and glucose cellular uptake [18]. In the same article, the authors showed that in mouse models of genetic and acquired obesity, chronic treatment with recombinant CT-1 lead to increased lipolysis, enhanced fatty acid oxidation, and stimulation of mitochondrial biogenesis as well as adipocytes shrinkage. Finally, treatment with CT-1 increased insulin-stimulated Akt and basal AMPK phosphorylation within the skeletal muscle, which might, respectively, explain the restored insulin responsiveness and increased fatty acid oxidation [18]. In line with the results of this important article, we have recently shown that high dose of CT-1 (10 nM) improved insulin responsiveness in cardiomyocytes through activation of AMPK and Akt [59]. Although controversies exist, short-term CT-1 administration might be considered as a potential treatment to prevent obesity, insulin resistance, and metabolic syndrome [55]. In fact, while acute administration of CT-1 might improve glucose metabolism and insulin resistance, chronic treatment might induce negative effects on the heart, arteries, and kidney [55]. Although a 10-day treatment with CT-1 did not cause any cardiac hypertrophy nor cardiac enlargement, a prolonged therapy with CT-1 (20 *μ*g/kg per day for 6 weeks) has been shown to increase myocardial dilatation and fibrosis, renal glomerular and tubule-interstitial fibrosis, arterial stiffness, and collagen content independently of blood pressure levels in CT-1-treated Wistar rats as compared to control vehicle. Therefore, chronic CT-1 administration has been indicated as a potential profibrotic approach particularly for the heart, vessels, and kidney [55]. In contrast to these effects observed with high dose of CT-1 (10 nM), we found that low dose of CT-1 (1 nM) is detrimental for the glucose metabolism in cultured cardiomyocytes [59]. These opposite activities of CT-1 on glucose metabolism in different organs could be partly explained by a dose-dependent effect. Accordingly, Zolk and coworkers described that chronically increased synthesis and release of CT-1 could accelerate contractile dysfunction, whereas acute administration of CT-1 could preserve contractility [60]. Thus, if confirmed, adverse effects of CT-1 in the myocardium and other organs have to be considered before starting a first-in-men clinical trial, mainly for chronic treatments.

## 6. Conclusion

Although promising protective activities have been shown for CT-1 against liver apoptosis [15], hepatocyte ischemic injury [16], and renal toxicity of iodinated contrast media [17], evidence from basic research studies indicates CT-1 to be a controversial molecule with potential opposite activities in obesity, insulin resistance, and related increased cardiovascular risk. CT-1 has been shown to promote cardiac hypertrophy, but also potentially restore insulin responsiveness. Therefore, it remains unclear whether CT-1 is a beneficial or deleterious cytokine in obesity. Furthermore, its role as a DAMP-triggering atherosclerotic inflammation is still debatable. The most relevant studies on CT-1 mediated effects *in vitro*, *in vivo,* and in humans on adipose tissue and obesity have been summarized in Table 1. Although CT-1 has been shown to activate several intracellular signaling pathways via its transmembrane gp130/LIFR receptor, further studies are needed to elucidate the different selectively triggered cellular functions and danger signals. Interestingly, very recently CT-1 has been described as key regulator of glucose and lipid metabolism in mice. However, it is also important to take into account the potential negative effects of CT-1 on the myocardium [42]. To clarify these controversial issues, the scientific community is waiting for the results of the first-in-men phase I, randomized, double blind clinical trial (NCT01334697) evaluating safety, tolerability, and early pharmacokinetics of the intravenous administration of recombinant human CT-1 versus placebo in healthy volunteers. Promising results in human beings might confer to this cytokine also a therapeutic potential.

## Figures and Tables

**Table 1:** Summary of the most relevant CT-1-mediated effects *in vitro*, *in vivo*, and in humans in adipose tissue and obesity.

Author [Ref.]	Year	Model	Exogenous treatment	Effects
*In vitro *				

Natal et al. [13]	2008	Murine 3T3-L1 preadipocytes versus differentiated adipocytes	Not applicable	Upregulation of CT-1 levels in differentiated adipocytes and in response to proinflammatory molecules

Zvonic et al. [14]	2004	Murine 3T3-L1 preadipocytes and differentiated adipocytes	Recombinant human CT-1 (0.02–2 nM)	Dose- and time-dependent activation and nuclear translocation of STAT1, -3, -5A, and -5B as well as ERK1 and -2

*In vivo*				

Zvonic et al. [14]	2004	7-week-old C57B1/6J mice	Recombinant human CT-1 at 0.5 microg/animal versus vehicle	Activation of MAPK, STAT-1, -3 in epididymal fat pads

López-Andrés et al. [55]	2012	Wistar rats	Treatment with rat recombinant CT-1 (20 *μ*g/kg per day till 6 weeks) versus vehicle	Chronic treatment with CT-1 increases fibrosis within heart vessel and kidney as compared to controls

Moreno-Aliaga et al. [18]	2011	CT-1 knockout versus wild-type mice under normal diet, high-cholesterol diet, or streptozotocin- (STZ-) induced diabetes	Treatment with rat recombinant CT-1 (0.2 mg/kg per day for 6–10 days) versus vehicle	CT-1 knockout mice develop obesity, insulin resistance, and hypercholesterolemia despite a reduced caloric intake as compared to wild type. Acute treatment with CT-1 decreased blood glucose in an insulin-independent manner as compared to vehicle. Chronic treatment with CT-1 treatment reduced food intake, enhanced energy expenditure, and induced white adipose tissue remodeling as compared to vehicle

Humans				

Natal et al. [13]	2008	Patients with metabolic syndrome (*n* = 43) versus healthy controls (*n* = 94)	Not applicable	Increased plasma levels of CT-1 in metabolic syndrome patients as compared to controls

Limongelli et al. [56]	2010	Triathletes versus matched controls (*n* = 20 per group)	Not applicable	During physical exercise, plasma levels of CT-1 were significantly increased as compared to levels at rest in triathletes

Jung et al. [58]	2008	White adolescents (aged 13 to 17 years) overweight (*n* = 37) versus normal weight controls (*n* = 35)	Not applicable	No increase in CT-1 plasma levels in overweight adolescents as compared to normal weight controls
